# Neuropsychiatric Phenotypes Produced by GABA Reduction in Mouse Cortex and Hippocampus

**DOI:** 10.1038/npp.2017.296

**Published:** 2018-01-24

**Authors:** Stefan M Kolata, Kazuhito Nakao, Vivek Jeevakumar, Emily L Farmer-Alroth, Yuko Fujita, Aundrea F Bartley, Sunny Zhihong Jiang, Gregory R Rompala, Robert E Sorge, Dennisse V Jimenez, Keri Martinowich, Yolanda Mateo, Kenji Hashimoto, Lynn E Dobrunz, Kazu Nakazawa

**Affiliations:** 1 Unit on Genetics of Cognition and Behavior, National Institute of Mental Health, National Institutes of Health, Department of Health and Human Services, Bethesda, MD, USA; 2 Department of Psychiatry and Behavioral Neurobiology, University of Alabama at Birmingham, Birmingham, AL, USA; 3 Division of Clinical Neuroscience, Chiba University Center for Forensic Mental Health, Chiba, Japan; 4 Department of Neurobiology, University of Alabama at Birmingham, Birmingham, AL, USA; 5 Department of Psychology, University of Alabama at Birmingham, Birmingham, AL, USA; 6 Lieber Institute for Brain Development and Department of Psychiatry and Behavioral Science and Department of Neuroscience, Johns Hopkins University School of Medicine, Baltimore, MD, USA; 7 National Institute on Alcohol Abuse and Alcoholism, National Institutes of Health, Department of Health and Human Services, Bethesda, MD, USA

## Abstract

Whereas cortical GAD67 reduction and subsequent GABA level decrease are consistently observed in schizophrenia and depression, it remains unclear how these GABAergic abnormalities contribute to specific symptoms. We modeled cortical GAD67 reduction in mice, in which the *Gad1* gene is genetically ablated from ~50% of cortical and hippocampal interneurons. Mutant mice showed a reduction of tissue GABA in the hippocampus and cortex including mPFC, and exhibited a cluster of effort-based behavior deficits including decreased home-cage wheel running and increased immobility in both tail suspension and forced swim tests. Since saccharine preference, progressive ratio responding to food, and learned helplessness task were normal, such avolition-like behavior could not be explained by anhedonia or behavioral despair. In line with the prevailing view that dopamine in anterior cingulate cortex (ACC) plays a role in evaluating effort cost for engaging in actions, we found that tail-suspension triggered dopamine release in ACC of controls, which was severely attenuated in the mutant mice. Conversely, ACC dopamine release by progressive ratio responding to reward, during which animals were allowed to effortlessly perform the nose-poking, was not affected in mutants. These results suggest that cortical GABA reduction preferentially impairs the effort-based behavior which requires much effort with little benefit, through a deficit of ACC dopamine release triggered by high-effort cost behavior, but not by reward-seeking behavior. Collectively, a subset of negative symptoms with a reduced willingness to expend costly effort, often observed in patients with schizophrenia and depression, may be attributed to cortical GABA level reduction.

## Introduction

Corticolimbic GABAergic dysfunction is implicated in the etiology of schizophrenia, depression, and related neuropsychiatric diseases (Benes and Berretta, 2001; Lewis *et al*, 2005; Nakazawa *et al*, 2012). For example, the level of *Gad1* mRNA encoding GAD67 (glutamic acid decarboxylase-67) falls below detectable levels in approximately 50% of parvalbumin (PV)-positive prefrontal cortex (PFC) interneurons in schizophrenia (Hashimoto *et al*, 2003). This has led to the hypothesis that GAD67 dysfunction, particularly in PV-positive *cortical* interneurons, may be implicated in development of schizophrenia (Lewis *et al*, 2005). However, several conditional *Gad1* deletion mouse lines have been tested for schizophrenia-related phenotypes with mixed results. For instance, a PV-neuron/*Gad1* homozygous knockout mouse line (Georgiev *et al*, 2016) was spontaneously hyperactive but showed normal sensorimotor gating and spatial working memory, and had increased mRNAs for *Pvalb*, *Bdnf*, *Kcns3* and *Gad2*. Since these mRNA are unaltered or decreased in patients with schizophrenia, it was suggested that PV neuron-selective GAD67 loss is not an upstream cause of schizophrenia (Georgiev *et al*, 2016). A conditional *Gad1* heterozygous knockout mouse line was also generated by crossing the floxed-*Gad1* line to a BAC(bacterial artificial chromosome)-Cre line, in which Cre is expressed not only in PV-positive interneurons but also in subsets of somatostatin- and calretinin-positive interneurons (Fujihara *et al*, 2015). This heterozygous KO mutant line showed MK-801 induced hyperlocomotion and sensorimotor deficits with a reduction in PV levels; conclusively demonstrating that haploinsufficiency of the *Gad1* gene in a subset of GABA neurons is sufficient to recapitulate schizophrenia-related phenotypes. In a third model, microRNA-mediated GAD67 knockdown mice exhibited sensorimotor deficits, as well as increased novelty-seeking and reduced fear extinction (Brown *et al*, 2015). The phenotypic discrepancies among these *Gad1* mutant mice are difficult to reconcile. However, one possibility is that it could be due to an insufficient reduction in cortical GABA in these mutant lines, or it could be attributed to unforeseen subcortical deficits of the GABA system due to the brain-wide manipulations.

In order to explore the impact of cortical GABA reduction following *Gad1* reduction, we genetically eliminated the *Gad1* gene from approximately 50% of cortical and hippocampal interneurons; a majority (70%) of which are PV-positive. This was accomplished by crossing a *loxP*-flanked *Gad1* mouse line (Chattopadhyaya *et al*, 2007) to a previously characterized *Ppp1r2*-Cre driver line (Belforte *et al*, 2010), in which Cre recombinase expression is largely confined to GABA neurons of the cortex and hippocampus. In this way we directly addressed which symptoms or phenotypes may be attributed to the lowered GABA levels in the cortex and hippocampus following GAD67 deficits.

## Materials and methods

Conditional *Gad1* homozygous knockout mutant mouse line was created by crossing a *loxP*-flanked *Gad1* line (Chattopadhyaya *et al*, 2007) to a *Ppp1r2*-Cre driver line (Belforte *et al*, 2010). Measurement of tissue GABA and glutamate content was conducted as previously described (Fujita *et al*, 2016). Slice physiology was conducted in acute medial PFC (mPFC) slices and hippocampal slices (Li *et al*, 2017). *In vivo* awake brain microdialysis was performed in anterior cingulate cortex (ACC) or nucleus accumbens (NAC) of freely moving mice using concentric microdialysis probes (CMA/7, CMA/Microdialysis, Solna, Sweden) (Tejeda *et al*, 2013). Briefly, dialysate samples were collected in a microdialysis test chamber three times (20-min bin each) prior to injecting with *d*-amphetamine (2.5 mg/kg; i.p.), before tail hanging, or before placing the animal in the operant conditioning chamber. Four additional samples (20-min bin each) were collected in the same chamber during and after amphetamine injection or tail hanging for 6 min. Dialysate samples were also collected from the animal in the operant chamber during progressive ratio (PR) schedule of nose-poking (20-min bin each, 80-min total). For dopamine and serotonin (5-HT) quantification, HPLC with electrochemical detection (Eicom HTEC-500 HPLC/EC System, Kyoto, Japan) with an PP-ODS2 column (Eicom) run in 20-μl samples with a mobile phase flow rate of 500 μl/min and an applied potential of +400 mV *vs* Ag/AgCl was used. To assess the epileptiform activity, *in vivo* local field potential (LFP) recordings from sensory cortex and hippocampus were conducted during a 10-min immobility session following animal’s cessation of exploratory behavior in a high-walled small circular box (10-cm in diameter) as previously described (Jinde *et al*, 2009). Male or female mutants and their floxed-littermate controls of 10–12 weeks of age, which were single-housed for at least 1 week prior to testing, were subjected to a behavioral test battery. Experimental procedures including the behavioral tests, drugs, and statistics are described in detail in Supplementary Information. All experimental procedures were in accordance with National Research Council guidelines for the care and use of laboratory animals, and were approved by the Institutional Animal Care and Use Committee at NIMH and at University of Alabama at Birmingham.

## Results

### Gad1 Ablation Leads to Reduced GABA Level and Altered GABA Transmission in mPFC and Hippocampus

**Figure 1:**
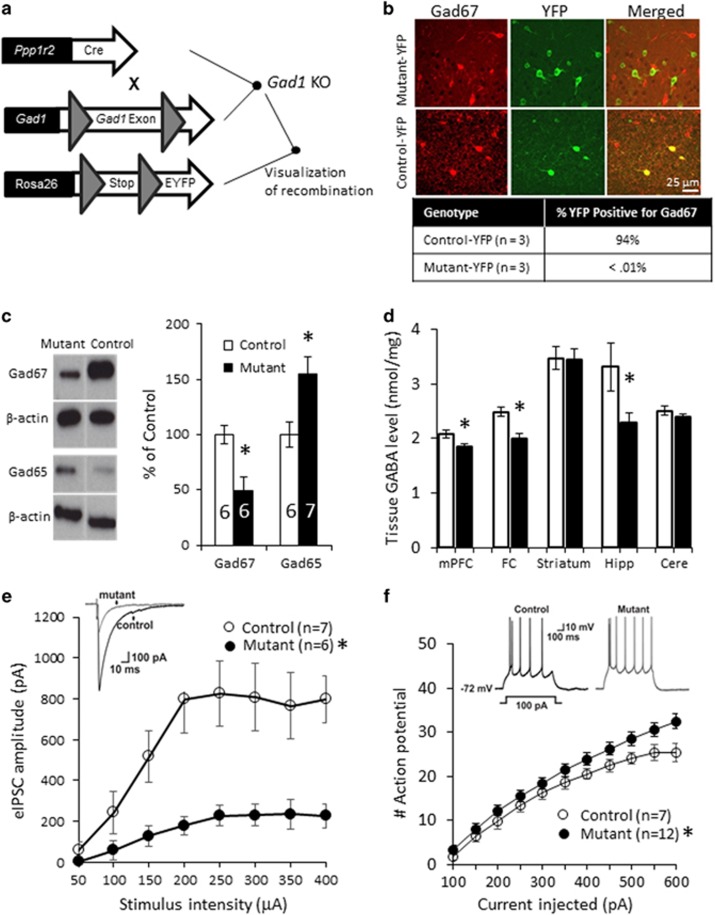
Outline of transgenic approach and validation of *Gad1* genetic ablation. (a) Schematic diagram showing generation of the conditional knockout of *Gad1*, *Ppp1r2*-Cre^+/-^:*Gad1*^loxP/loxP^, referred to as mutant and of the triple transgenic mouse that allows visualization of Cre-targeted cells by enhanced YFP (EYFP), *Ppp1r2*-Cre^+/-^:*Gad1*^loxP/loxP^: Rosa26 ^loxP-Stop-LoxP^ – EYFP^+/-^, referred to as YFP-mutant. (b) Double immunolabeling in the mPFC of GAD67 (red) and YFP (green) in mPFC of 10-week-old mice showing the YFP cells positive for GAD67. The table shows %YFP cells positive for GAD67 (out of 135 cells in the controls; 132 cells in the mutants in mPFC) in both genotypes demonstrating GAD67 deletion virtually all YFP-positive cells in mutant mice. (c) Representative western blot bands and their quantification (in graph) showing that GAD67 protein is reduced in somatosensory cortical tissue homogenates of 10-week-old mutants (t(10)=3.09, *p*<0.01) while GAD65 is increased (t(11)=3.04, *p<0.01). (d) Tissue GABA content in the control floxed mice (white, *n*=14) and mutant mice (black, *n*=13). GABA level is lower in mPFC (t(25)=2.69, *p=0.0013), frontal cortex (FC, t(25)=4.07, *p=0.0004) and hippocampus (Hipp, t(25)=2.09, *p=0.046), but not in striatum (t(25)=0.12, *p*=0.91) or cerebellum (Cere, t(25)=1.03, *p*=0.31). (e) *Gad1* ablation in GABAergic interneurons reduces the amplitude of evoked IPSCs in the anterior cingulate cortex (ACC). Plot of evoked IPSC amplitudes in slices from control and mutant mice (two-way ANOVA, F(1,88)=64.5 for genotype main effect, *p<0.001). Inset: Representative traces from control and mutant groups showing the difference in amplitudes at the same stimulation intensity. (f) Pyramidal neurons in mutant mPFC are more excitable compared to controls. Summary graph of the firing frequency of pyramidal cells with the mutant group exhibiting significantly higher number of action potentials in response to somatic current injection (two-way ANOVA, F(1,187)=27.1 for genotype main effect, *p<0.001). Inset: Representative recordings showing the firing patterns of pyramidal neurons, for a current injection of 100 pA. All Data are mean±SEM; animal number or cell number *n* is indicated in parentheses or plot bars.

Homozygous *Gad1*loxP/loxP;*Ppp1r2*-cre progeny (*Ppp1r2*-cre^+/−^;*Gad1*^loxP/loxP^, hereafter simply mutant) were viable and fertile, with no gross morphological abnormalities revealed by Nissl staining (data not shown). We first assessed the extent of genetic *Gad1* ablation by crossing the conditional *Gad1* KO mutants further to a floxed-EYFP line (Figure 1a). Immunoreactivity of GAD67 encoded by *Gad1* was absent in nearly all the Cre-targeted YFP-positive cells in the cortex and hippocampus of the mutant mice at 10 weeks old (see Figure 1b). Tissue homogenate GAD67 levels in somatosensory cortex were also reduced to roughly 50% (Figure 1c), which is consistent with the evidence that ~50% of cortical interneurons express Cre recombinase (Belforte *et al*, 2010). On the other hand, protein level of GAD65, the enzyme responsible for a minority of GABA synthesis, were increased in somatosensory cortex of the mutants (Figure 1c), presumably due to compensation. Nonetheless, tissue GABA levels, measured by HPLC, were reduced in mPFC, frontal cortex (a frontal portion of neocortex), and hippocampus of the mutant mice at 11–13 weeks old (Figure 1d). In contrast, no GABA level reduction was detected in striatum or cerebellum where Cre expression is negligible (Belforte *et al*, 2010). Since no change in tissue L-glutamate levels was detected in any tissues examined, the GABA/glutamate ratio was also diminished in mPFC, frontal cortex, and hippocampus of the mutant mice (Supplementary Figure S1A).

Next, we compared the GABAergic synaptic transmission to pyramidal neurons in the layer 2/3 of ACC by applying electric stimulation of different intensities (from 50 μA to 400 μA) *ex vivo*. The stimulation delivered in the vicinity of the patched neuron in the presence of glutamate blockers (CNQX and D-APV) produced an inhibitory postsynaptic current (IPSC) response which initially increased linearly until approaching a plateau in both groups (Figure 1e). However, the response in the mutants was smaller at all stimulus intensities, suggesting impaired efficiency of GABAergic synaptic transmission following *Gad1* knockout. We also investigated whether the *Gad1* knockout causes changes in the excitability of layer 2/3 pyramidal neurons in ACC. By injecting a series of hyper- and de-polarizing current steps into the soma of the recorded neuron from resting membrane potential, action potentials were evoked and the membrane properties were measured. All cells recorded showed regular spiking and adapting action potentials. Threshold, action potential half-width and amplitude, fast-AHP (after hyperpolarization), input resistance and membrane time constant did not vary significantly between genotypes (Supplementary Figure S1B). However, mutant neurons were more excitable as indicated by the higher number of action potentials fired (Figure 1f), indicating that the pyramidal neurons in mutant mice may be disinhibited due to impairment of GABAergic transmission. A similar decrease in evoked IPSC amplitudes was also observed in CA1 pyramidal neurons, as well as an increase in the evoked action potential number, supporting the GABA level reduction in the hippocampus (Supplementary Figures S2 and S3 and see Supplementary Discussion).

### Avoidance of Center/light Arena Exploration, Social and Sexual Withdrawal and Higher Immobility during Effortful Behaviors

**Figure 2:**
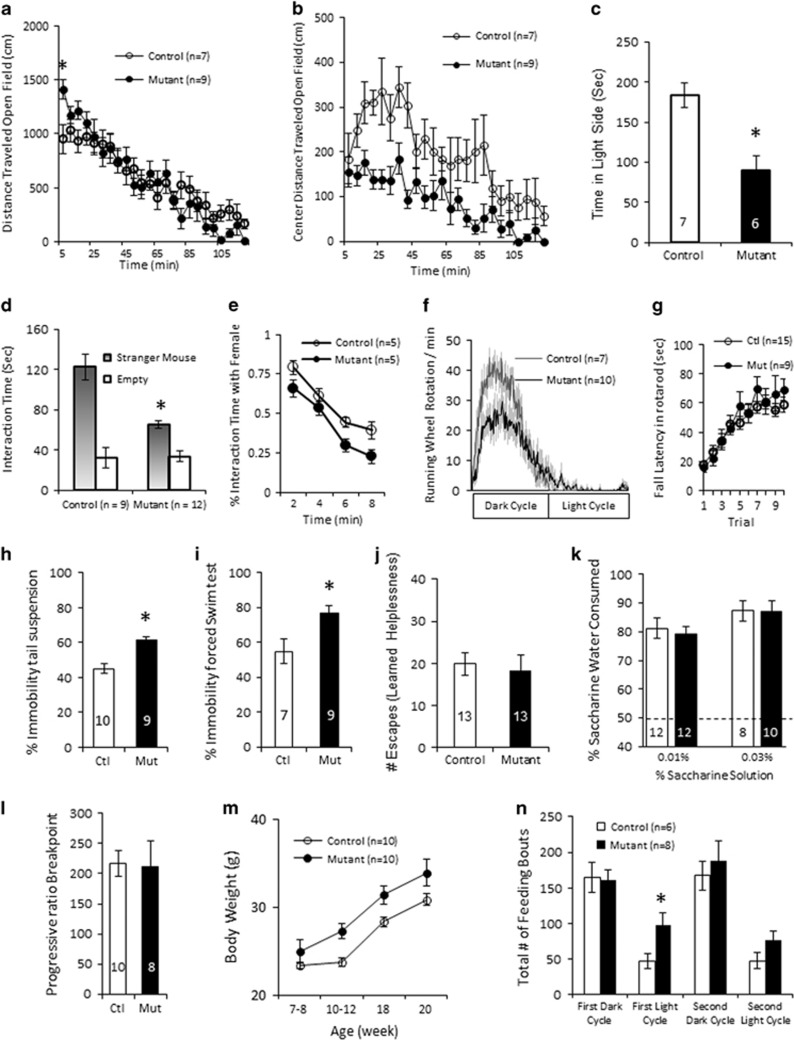
Mutants exhibited a subset of negative symptom-like behavioral deficits. (a) Locomotor behavior in the open field did not differ between the genotype overall (F(1,23)=.33, *p*=0.57) but there the mutant mice displayed novelty induced hyperlocomotion in the first 5 min (*post-hoc* planned comparisons, F(1,14)=5.18, *p<0.05). (b) Mutant mice spent significantly less time in the center of the open field (F(1,23)=13.74, *p*<0.01). (c) Mutant mice spent less total time in the light side of the light/dark box (t(11)=3.7, *p<0.01). (d) Overall social interaction time with a novel mouse in the sociability test was significantly reduced in the mutants (F(1,19)=8.143, *p*<0.02) and there was a significant interaction between genotype × novel/empty cage interaction (F(1,19)=57, *p<0.01). (e) Over 8-min period, mutant mice spent significantly less time interacting with the sexually receptive female mice (F(1,9)=17, *p*<0.01). (f) Mutant mice differed significantly in their motivated wheel-running behavior (genotype × time interaction: F(1,120)=2.68, *p*<0.001) and their peak wheel running activity was attenuated (*post-hoc* planned comparisons, F(1,15)=1.23, *p*<0.02). (g) No difference in rotarod performance was observed between mutant (Mut) and control (Ctl) mice (12–19 weeks of age), repeated measures ANOVA F(1,836)=0.47, *p*=0.50. (h) Male mutants showed increased immobility duration in tail suspension, t(17)=4.51, *p=0.0003). (i) Male mutants showed increased immobility duration in forced swim chamber (t(14)=−2.77, *p=0.015). (j) No genotypic differences in the number of escapes in the learned helpless task (t(23)=0.56, *p*=0.58). (k) No genotypic differences in their preference for saccharin sweetened water at 0.01% (t(22)=0.48, *p*=0.64) and 0.03% (t(16)=0.02, *p*=0.98) solution. (l) No genotypic difference in breakpoint achieved in PR session following the acquisition training in fixed ratio 1 (FR1) and FR5 schedule of reinforcement to obtain chocolate pellets. t(16)=0.14, *p*=0.89. (m) Mutant mice showed increased weight gain (F(1,16)=3.7, *p*<0.05). (n) In the 48-h homecage scan, mutants showed increased feeding time during their first light cycle (600–1800 hours) (t(12)=1.8, *p<0.05). Data are mean±SEM; animal number *n* is indicated in parentheses or plot bars.

To assess the impact of cortical GABA reduction on murine behavior, we subjected male mice to a behavioral test battery. In a novel open field, the mutants displayed elevated, novelty-induced activity relative to the controls during the first 5 min (Figure 2a); however subsequently, the mutants spent less time in the center of the open field (Figure 2b). The mutant mice also spent less total time in the light side of light/dark box (Figure 2c), suggesting an increased anxiety or decreased risk-taking. In the three-chamber sociability test, the mutants showed a decrease in time spent exploring the stranger mouse cage *vs* the empty cage, which is indicative of social withdrawal (Nadler *et al*, 2004) (Figure 2d). The male mutant mice also spent less time interacting with super-ovulated female mice over an 8-min period (Figure 2e). Similarly, male mutants at 15-week-old showed reduced mating efficiency with super-ovulated female B6 wild-type mice (4/24 mutants mated *vs* 17/17 controls, judged by copulatory plug formation on the following day). In the voluntary wheel running behavior, the peak wheel running activity of the mutants was reduced (Figure 2f). This is unlikely to be caused by the motor dysfunction, because mutants’ voluntary locomotion (Figure 2a) and motor coordination in the rotarod test (Figure 2g) were normal. Similarly, the mutant mice used less nestlet material when tested in an overnight nest-building test (control: *n*=10, unused nestlet (out of 1.9 g), *avg.*=0; mutant: *n*=10, *avg.*=1.2(±0.5), data not shown). These results suggested that the mutants show increased socially/sexual anxiety and/or are less willing to engage in effortful behavior.

To further assess if the mutants were impaired in effortful behavior engagement, immobility was measured in the tail suspension (Figure 2h) and forced swim tests (Figure 2i). The mutants showed heightened immobility in the both tasks compared to the controls. However, no genotypic differences were observed in the learned helplessness test, in which animals are exposed to unavoidable shock and later are trained to escape from the shock (Shirayama *et al*, 2002) (Figure 2j). These results suggest that the mutant mice do not display a depressive-like phenotype. We further tested a sweet-taste preference in male mice that were placed in individual cages and offered choice between 0.01% or 0.03% saccharin or water for 24 h (Figure 2k). Both control and mutant mice drank more saccharin than water, and there was no difference between genotypes. Using a different cohort of animals, mice were trained to respond on a nose-poke aperture of an operant conditioning chamber, with fixed ratio (FR1 and FR5) schedule of reinforcement, and all the animals of both genotypes reached the acquisition criteria (see Supplementary Method). Subsequently, the mice were required to respond on a progressive ratio (PR) schedule of reinforcement (Richardson and Roberts, 1996) for food. We found that the average break points (Figure 2l), the total nose-poke number (Supplementary Figure S4A), and average rate of nose-poking during the session (Supplementary Figure S4B) were all comparable between the genotypes. The results were consistent with a recent report showing that reducing mPFC GABA transmission exerts little effect on PR responding to sucrose reward in rats (Piantadosi *et al*, 2016). These findings also suggested that mutant mice are normal in hedonic (saccharine preference test) and reward reinforcement (PR responding) behavior, but exhibit effort avoidance when reward provided is negligible, such as in the forced swim test, tail suspension test, and spontaneous wheel running. Interestingly, the mutant mice showed increased body weight gain after 8 weeks of age (Figure 2m), and in the 48-h-homecage scan, they showed increased feeding time during their expected sleep (light) cycle at 10–12 weeks of age (Figure 2n), despite no other significant activity changes over a 24-h period (Supplementary Figure S4C).

### No Deficits in Cognitive or Positive Symptom-like Phenotypes of Schizophrenia

**Figure 3:**
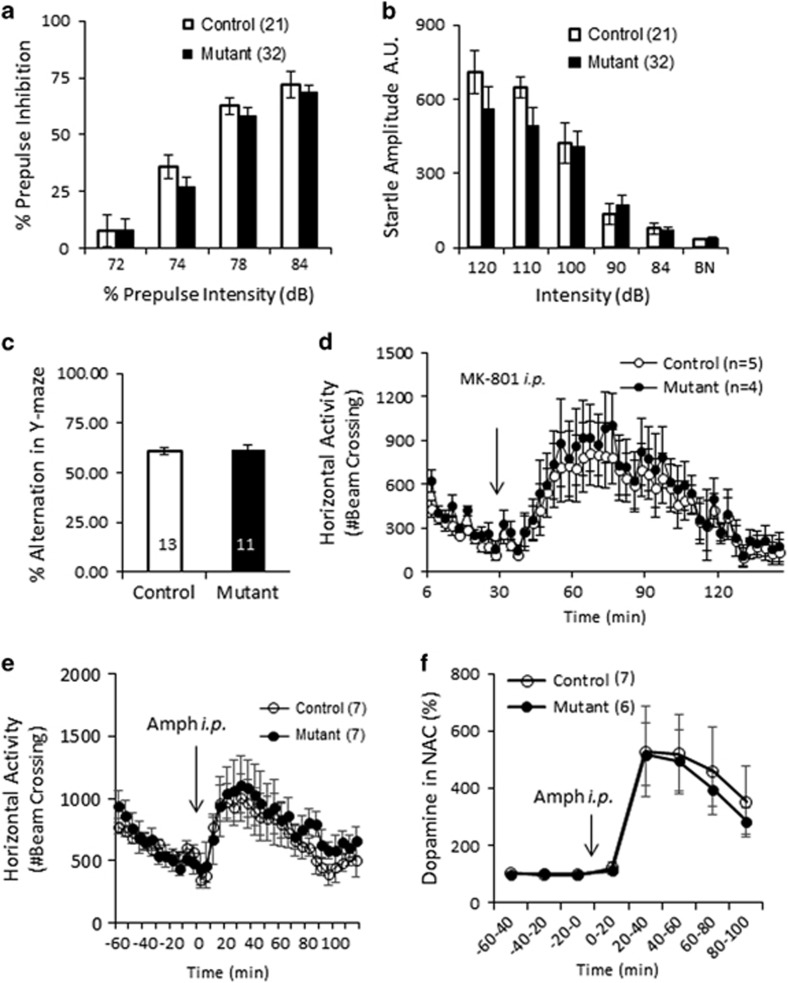
No typical schizophrenia-related phenotypes in mutant mice. (a) No difference was detected between the genotypes in the pre-pulse inhibition of the startle reflex (F(1,51)=0.9, *p*=0.34), and (b) no differences in the startle reflex magnitude (F(1,51)=1.2, *p*=0.27). (c) There were no differences in the % alternation in the spatial Y-maze working memory task (t(22)=−0.30, *p*=0.76). (d) MK-801 (0.3 mg/kg, i.p.) showed an increase in the horizontal activities (# beam cross) in the open field. No difference in altered responsiveness to MK-801 (F(1,49)=2.6, *p*=0.11). (e) Amphetamine (2.5 mg/kg, i.p.) showed an increase in the horizontal activities (# beam cross) in the open field. No difference between the genotypes in the horizontal activities before (repeated measures ANOVA, F(1,132)=1.49, *p*=0.14) and after the drug treatment (repeated measures ANOVA, F(1,276)=0.30, *p*=0.99). (f) No difference between the genotypes in the baseline dopamine levels in NAC lateral shell (t(11)=0.48, *p*=0.65) before the amphetamine treatment. No difference between the genotypes in amphetamine (Amph)-induced dopamine increase in NAC lateral shell (repeated measures ANOVA, F(1,44)=0.10, *p*=0.98). Data are mean±SEM; animal number *n* is indicated in parentheses or plot bars.

Prepulse inhibition (PPI) is consistently impaired in patients with schizophrenia and is considered a read-out of impaired sensorimotor gating (Braff *et al*, 2001). In 10–12 week-old mutant mice, there was neither a deficit in the degree of PPI (Figure 3a) nor an alteration in the startle reflex responses (Figure 3b). In the continuous alteration Y-maze task, the mutants showed no difference in either the number of alternations (Figure 3c) or in the total number of arm entries (*avg.* control=30.9 (±2.06); *avg.* mutant=35.8 (±2.44); *p*>0.05, data not shown), which is suggestive of no spatial working memory impairments. In the NMDA antagonist MK-801-induced hyperlocomotion test, a model psychotomimetic response (van den Buuse, 2010), the mutants showed no differences in the extent of locomotor hyperactivity with a moderate dose (0.3 mg/kg) of MK-801 (Figure 3d). Amphetamine-induced hyperlocomotion, another measure of psychotomimetic response (van den Buuse, 2010), was also comparable to the control mice (Figure 3e). Indeed, *in vivo* awake microdialysis revealed no genotypic difference in an extracellular dopamine release in NAC lateral shell in response to systemic amphetamine treatment (Figure 3f and Supplementary Figure S5B), whereas patients with schizophrenia are known to increase striatal dopamine by amphetamine administration (Laruelle *et al*, 1999). Baseline dopamine levels in NAC were also unaltered in the mutants (control: 0.83 nM±0.04; mutant: 0.76 nM±0.1; t(9)=0.4; *p*=0.69). Taken together, these results suggested that male mutant mice do not display cognitive or positive symptom-like phenotypes of schizophrenia, consistent with the conclusion reported for PV-neuron/*Gad1* KO mice (Georgiev *et al*, 2016).

### Female Mutant Mice with Less Epileptiform Discharges Shared Behavioral Deficits

**Figure 4:**
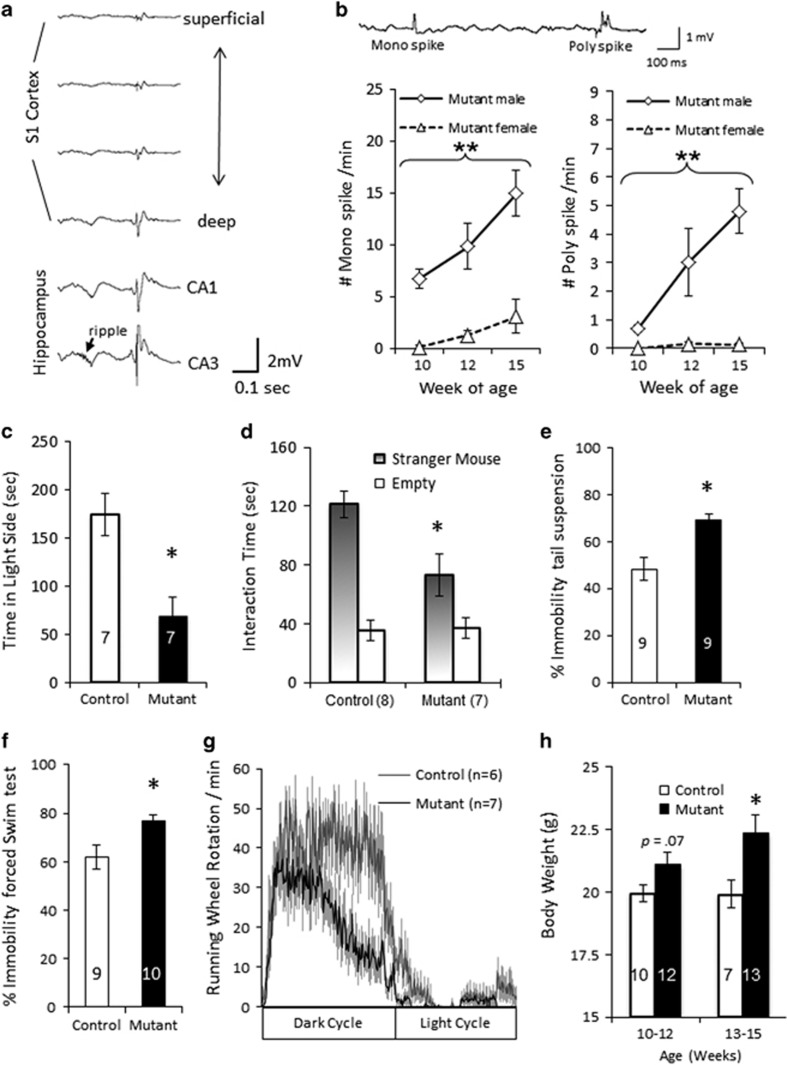
Female mutants at 10–12 weeks of age, with negligible epileptiform activity, shared similar magnitude of behavioral deficits. (a) Representative example of LFPs from primary somatosensory (S1) cortex and hippocampal areas CA1 and CA3 of 10-week-old mutant male mouse while the animal was held immobile in a small chamber (10 cm in diameter). Mutant mice displayed interictal discharges which were synchronized across cortical and hippocampal electrodes, with the higher amplitudes in the ripple-carrying hippocampal electrodes. (b) The number of mono-spikes detected in S1 cortex of 10-week-old male mutant mice was higher than 10-week-old female mutant and 10-week-old control male mice during the 10-min immobility period (F(1,16)=8.56, *p*=0.0029). The number of poly-spikes in 10- and 15-week-old male mutant mice was also much higher than 10-week-old mutant female mice during 10-min immobility period (F(1,16)=7.94, *p*=0.004). Repeated measures ANOVA, **p<0.01. (c) Female mutant mice spent less time in the light side of the light/dark chamber than did their female floxed-control mice (t(12)=3.6, *p<0.01). (d) Social interaction time with a novel mouse (Stranger) in the sociability test was significantly reduced in female mutant mice (*post-hoc* planned comparisons: F(1,12)=6.2, *p<0.05). (e) Female mutants showed increased immobility duration in tail suspension (t(16)=−4.01, *p=0.001). (f) Female mutants showed increased immobility duration in forced swim chamber (t(17)=−2.76, *p=0.013). (g) Female mutants differed from the female controls in home-cage wheel-running behavior (genotype × time: F(1,120)=2.1, *p*<0.05). (h) Female mutant mice were heavier than female controls at 13–15 weeks old (*p<0.05), while they tended to weigh more already at 10–12 weeks of age (*p*=0.07). Data are mean±SEM; *n* is indicated in parentheses or plot bars.

LFP recordings were conducted simultaneously in somatosensory cortex and hippocampus of mutant male and female mice and control mice while they were in an immobile state in a small circular enclosure (10 cm diameter). Male mutants (*n*=5), but not floxed-*Gad1* control male mice (*n*=5, see Supplementary Figure S6), at 10 weeks of age showed the presence of interictal discharges, the frequency of which became progressively worse with age (Figure 4a and b). The epileptiform discharges were mostly of a larger magnitude in the hippocampus than those in the cortex, and the onset of the discharges was synchronized across the electrodes (Figure 4a). Unexpectedly, these epileptiform discharges were barely detected in female mutant mice (*n*=5) until 15 weeks of age. After recording in the immobile state, the mice were forced to explore in a low-walled linear track (length, 72 cm; width, 8 cm). During the exploratory behavior, epileptiform discharges were not observed in male (*n*=5) or female (*n*=5) mutant mice, even at 15 weeks of age (data not shown).

To evaluate whether the observed male mutants’ behavioral changes in Figure 2 are affected by their epileptiform activity, we subjected the female mutant mice at 10–12 weeks old to the behavioral tests because at this age they lacked the interictal discharges. Similar to the male mice, the female mutants showed avoidance of risk area exploration (Figure 4c), social withdrawal (Figure 4d), higher immobility in both tail suspension (Figure 4e) and forced swim tests (Figure 4f), decreased voluntary wheel running (Figure 4g), and body weight gain (Figure 4h). They also showed nest building impairments (control: *n*=8, *avg.* unused nestlet (out of 1.9 g)=0; mutant: *n*=8, *avg.*=1.1(±0.39)). These results indicated that behavioral phenotypes in male mice are shared with females, and are thereby unlikely to be attributed to males’ epileptiform activity.

### ACC Dopamine Release Impaired in Tail Suspension but Normal in PR Responding

**Figure 5:**
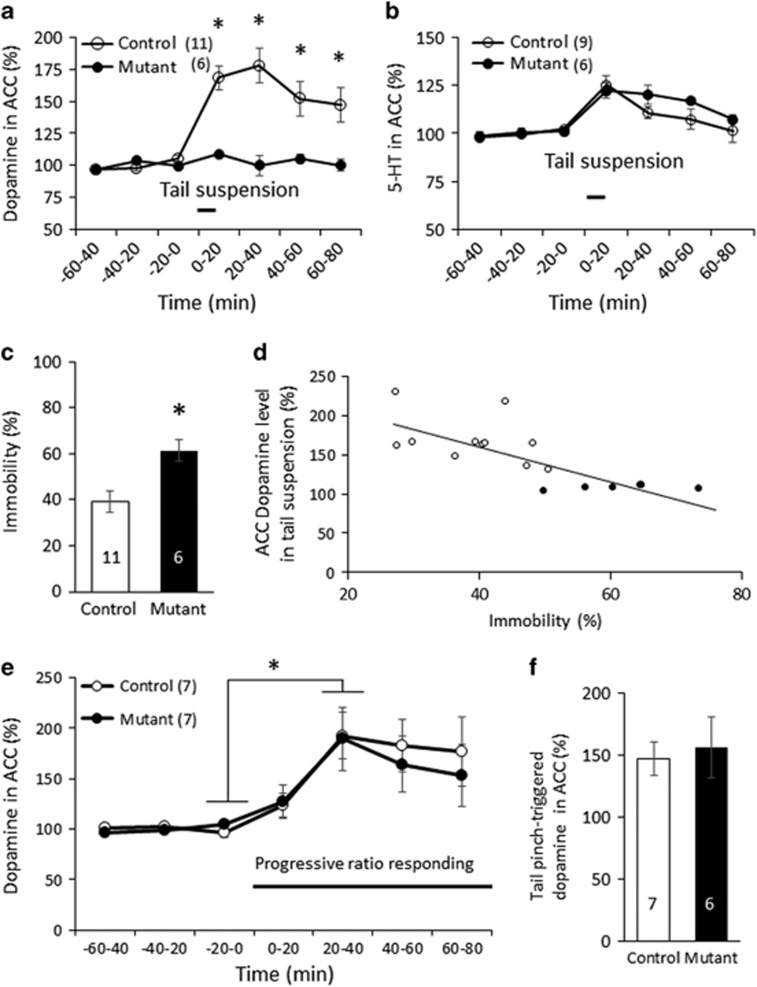
Tail suspension-triggered dopamine release observed in ACC was deficient in mutant mice. (a) *In vivo* microdialysis revealed that control mice (11 floxed mice (5 males and 6 females) at 15–20 weeks of age) augmented the dopamine level in ACC by 1.7-fold from the baseline by tail suspension for 6 min (paired *t*-test between −20–0 min and 0–20 min, t(10)=−7.99, *p*=1.18E-05), regardless of gender (male mice, t(4)=−9.02, *p*=0.0008; female mice, t(5)=−5.5, p=0.002). While the mutants (6 mice at 14–18 weeks of age) also showed small dopamine release (paired *t*-test between –20–0 min and 0–20 min, t(5)=−3.49, *p*=0.017), robust difference in tail hanging-triggered dopamine release was observed between controls and mutants (repeated measures ANOVA, F(1,45)=2.44, *p*=0.07, *post-hoc* planned comparisons, at 0–20 min, F(1,15)=22.1, *p=0.00028; at 20–40 min, F(1,15)=16.1, *p=0.0011; at 40–60 min, F(1,15)=6.32, *p=0.023; at 60–80 min, F(1,15)=6.03, *p=0.026). Before tail suspension, there was no genotypic difference in the baseline dopamine levels in ACC (0.66 nM for controls and 0.75 nM for mutants (t(15)=−0.34, *p*=0.74). (b) Tail suspension increased 5-HT levels by 1.2-fold from the baseline in ACC both mutants (6 mice at 14–18 weeks of age; paired *t*-test between –20–0 min and 0–20 min, t(5)=−4.79, *p*=0.0049) and controls (9 mice (5 males and 4 females) at 15–20 weeks of age; paired *t*-test between –20–0 min and 0–20 min, t(8)=−4.31, *p*=0.002). However, there was no genotypic difference in the degree of 5-HT elevation (repeated measures ANOVA, F(1,39)=0.46, *p*=0.71). Before the tail suspension, there was no genotypic difference in the baseline 5-HT level in ACC (0.63 nM for controls and 0.68 nM for mutants. (t(13)=−0.19, *p*=0.85). (c) Mutant mice during dialysate sampling showed increased immobility during 6-min tail suspension, compared to the control mice (t(15)=−5.37, *p=0.0002). (d) A robust inverse correlation was observed between ACC dopamine increase and tail suspension-elicited immobility across genotypes (Pearson’s correlation coefficient, *r*=−0.75, *P*=0.00053). (e) *In vivo* microdialysis revealed that both mutants and controls increased ACC dopamine levels during PR schedule of reinforcement (paired *t*-test between –20–0 min and 20–40 min, control: t(6)=−4.411, *p=0.0045, mutant: t(6)=−2.83, *p=0.029). No difference in the extent of dopamine increase observed between genotypes (repeated measures ANOVA, F(1,36)=0.181, *p*=0.91). (f) No difference in the extent of ACC dopamine release following tail pinch for 1 min between genotypes in the first 20-min bin (t(11)=−0.34, *p*=0.74). Data are mean±SEM; *n* is indicated in parentheses or plot bars.

Among mutant behavioral phenotypes, the most puzzling finding was a heightened immobility in the tail suspension and forced swim tests, despite that effort-related motivation was normal which was assessed by the PR nose-poking. Actually, prefrontal GABA transmission blockade in rats has been suggested to alter the cost/benefit decision making (Paine *et al*, 2015; Piantadosi *et al*, 2016), while the PR responding task was minimally affected (Piantadosi *et al*, 2016). Furthermore, ACC dopamine has been suggested to play a major role in effort allocation (Bailey *et al*, 2016; Kurniawan *et al*, 2011; Miller *et al*, 2013; Salamone *et al*, 2016). 5-HT also appeared to play a role in overcoming effort cost (Meyniel *et al*, 2016). We sought to examine whether ACC dopamine level could be altered, concordant with the behavioral deficit. First, we measured extracellular levels of dopamine and 5-HT in the ACC before and after the tail suspension by *in vivo* awake brain microdialysis (Supplementary Figure S5A). Prior to tail suspension, baseline levels of dopamine and 5-HT of the mutant mice were both comparable to the controls. Hanging control mice, regardless of gender, by the tail for 6 min significantly increased extracellular dopamine in ACC by ~70% at least for 80 min followed by a gradual decline (Figure 5a). In contrast, hanging-triggered dopamine release was robustly impaired in the ACC of mutant mice. In the same dialysate samples collected, 5-HT level also modestly increased in both genotypes by tail hanging, suggesting 5-HT release is normal in the mutants (Figure 5b). Higher immobility of the mutants compared to controls was confirmed in this cohort of animals (Figure 5c). Per-animal analysis revealed a significant inverse correlation between immobility (%) and increased ACC dopamine fold-changes in combined genotypes (Figure 5d). Indeed, mutants with impaired dopamine release all showed higher immobility. Interestingly, no robust increase in dopamine (Supplementary Figure S7A) or 5-HT (Supplementary Figure S7B) concentrations was observed in the NAC (see Supplementary Figure S5) during tail suspension.

Tonic dopamine release in mPFC may also be triggered by cognitive demand (Karakuyu *et al*, 2007; Stefani and Moghaddam, 2006), aversion (Mantz *et al*, 1989; Pignatelli and Bonci, 2015) and stress (Holly and Miczek, 2016). We evaluated the ACC dopamine release during PR responding task in which the mutants showed comparable PR breakpoints to the controls (Supplementary Figure S7D). Simultaneous *in vivo* microdialysis experiment (Supplementary Figure S5A) revealed a robust reinforcement-triggered dopamine release in ACC of these mutant mice (Figure 5e and Supplementary Figure S7C). We also assessed the ACC dopamine release induced by tail pinch for 1 min. This stressful and noxious stimulus also elicited the ACC dopamine release in the mutants (Figure 5f). Therefore, cortical GABA reduction-mediated dopamine release deficits appeared to occur in a particular circuity in ACC by which effortful behavior triggers dopamine release, such as in the tail suspension.

## Discussion

We examined whether cortical GABA level reductions could precipitate psychiatric-like phenotypes in mice, in which *Gad1* was genetically ablated in ~50% of cortical GABA neurons. The mutant mice grew normally but demonstrated a cluster of effort-based behavior deficits, including decreased home-cage wheel running and increased immobility in both tail suspension and forced swim tests. *In vivo* microdialysis from awake behaving animals revealed that tail suspension triggered the extracellular release of dopamine in ACC of the control mice. However, with increased immobility, the mutant mice showed a robust deficit in effort-triggered dopamine release in AAC. Interestingly, the mutants had normal release of dopamine in ACC during PR responding, a classical reward reinforcement task. These results suggest a novel association from cortical GABA reduction to impaired high-effort cost behavior, but not to reward-seeking behavior, through ACC dopamine deficiency. These results also shed light on the role of cortical GABA reduction in a subset of negative symptom-like phenotypes of major psychiatric disorders.

Of particular significance in the present study is that cortical GABA reduction results in a deficit in effort-triggered dopamine release in the ACC during the tail suspension test. The ACC is known to be critically involved in effort-based decision making (Bailey *et al*, 2016; Kurniawan *et al*, 2011; Miller *et al*, 2013; Salamone *et al*, 2016). For example, lesions or inactivation of the ACC results avoidance of previously preferred high-effort options, leading to a higher likelihood of choosing low-effort options (Floresco and Ghods-Sharifi, 2007; Hosking *et al*, 2014; Rudebeck *et al*, 2006; Walton *et al*, 2003; Walton *et al*, 2009). In particular, ACC dopamine appears to modulate the willingness of animals to exert physical effort, because dopamine depletion via 6-hydroxydopamine lesions (Schweimer *et al*, 2005) (but see Walton *et al*, 2005) or dopamine receptor blockade (Aly-Mahmoud *et al*, 2017; Schweimer and Hauber, 2006; Wang *et al*, 2017) in ACC resulted in effort avoidance during cost-benefit decision making. Consistent with such previous reports, we found, in control mice that tail hanging increases dopamine concentration in the ACC. In addition, there was an inverse correlation observed between the degree of immobility and ACC dopamine fold-increase. Notably, all the mutant mice tested exhibited high immobility scores in the tail suspension test, and showed little increase in ACC dopamine in the same test. These results suggested that the effort required to escape from tail hanging is associated with ACC dopamine increase. Since PR responding to food, a classic measure of effort-related motivation, and ACC dopamine release during this task was both comparable to that in the control mice, ACC dopamine appears to not always be responsible for all types of cost-benefit decision making, as previously reviewed (Walton *et al*, 2007). Indeed, ACC dopamine receptor inactivation has recently been shown to impair a rat nose-poking task that requires large effort with less reward, but the similar nose-poke task that allowed the animal to effortlessly perform nose-poking to obtain reward was not impaired (Wang *et al*, 2017). A plausible idea to explain these results is that GABA reduction preferentially impairs the effort-based behavior which requires considerable effort with little benefit, through ACC dopamine release deficit in ACC. In other words, the ACC dopamine release mechanism would be disturbed by cortical GABA reduction when the release is triggered by effortful behavior with little reward, such as tail suspension. Since such low-benefit high-cost task is inherently stressful, it is plausible that the ACC dopamine release deficit by tail suspension could rather be due to altered stress sensitivity of the mutants. Actually, stress is known to increase in ACC dopamine release (Holly and Miczek, 2016). However, normal ACC dopamine release triggered by tail pinch (ie, aversive stressful stimulus) in the mutants might preclude this possibility. Conversely, despite GABA reduction, ACC dopamine release would be unaffected when triggered presumably by reward-prediction or reward-seeking in PR responding task. Accordingly, it is conceivable that multiple dopamine release mechanisms may exist in the ACC and that GABA reduction following Gad67 KO would impair the release mechanism triggered by high-effort cost behavior, but not by reward-seeking behavior. Further research is warranted to decipher the mechanism underlying GABA level-dependent cortical dopamine release.

In the present study, the impact of *Gad1* ablation appeared after young adulthood, as evidenced by the development of epileptiform discharges in male mutants after 10 weeks of age which gradually increased with age. This slow development could be due to the compensatory increase in GAD65 protein, which is also reported in other *Gad1* KO mouse models (Fujihara *et al*, 2015; Georgiev *et al*, 2016). These aberrant LFP wave-forms in the cortex could conceivably underlie the behavioral deficits seen in the mutants and this possibility cannot be totally discounted. However, this interpretation is not entirely parsimonious with the data as female mutants before 13 weeks of age did not show such epileptiform activity but still showed similar behavioral deficits as male mutant mice. It is puzzling that the female mutants appear to be more resilient to epileptiform activity. This mutant mouse line could be a useful model to study the differential role of sex hormones in epilepsy as testosterone has been suggested to exacerbate temporal lobe epilepsy (Mejias-Aponte *et al*, 2002).

A somewhat puzzling finding in this study was that the spatial Y-maze alternation task to assess spatial working memory was normal in mutants. GABAergic transmission deficits are often linked to cognitive deficits, including working memory (Tse *et al*, 2015). Association between GABA content in dorsolateral PFC and working memory load, but not its maintenance, has been demonstrated in human subjects (Yoon *et al*, 2016). The mPFC GABA_A_ receptor blockade with bicuculline has also been shown to cause an increase in choice latency in the eight-arm spatial working memory task; however, choice accuracy was normal (Enomoto *et al*, 2011). Perhaps, testing with such highly elaborate rodent cognitive tasks may uncover the mutant cognitive phenotype.

A limitation of the present study is that GABA reduction in other brain regions may also impact on the behavioral phenotypes, because GABA reduction is not limited to the ACC. For example, both orbitofrontal cortex and prelimbic cortex may differentially affect a part of the cost–benefit analysis process, in particular, reward-learning and reward-valuation processes, which allows motivation to energize behavior (Bailey *et al*, 2016; Barch *et al*, 2016). Indeed, prelimbic cortex lesion in mice has been shown to decrease the breakpoints in the PR schedule (Gourley *et al*, 2010). Although normal PR responding by our mutants suggests that involvement of prelimbic GABA reduction in effort avoidance is unlikely, further studies are warranted to determine the role of GABA in each brain area involved in effort-based behavior.

Our results may have clinical implications in the elucidation of the role of reduced GABA levels in neuropsychiatric disorders, particularly a subset of negative symptoms. Although lower cortical GABA levels have been reported in several neuropsychiatric disorders, including schizophrenia and depression (Egerton *et al*, 2017; Schur *et al*, 2016), it remains to be determined whether cortical GABA reduction produces common deficits across the current diagnostic categories or whether it is uniquely associated with different facets of each disorder. Since the reduced effort allocation has also been reported in both schizophrenia and depression (Barch *et al*, 2016; Green *et al*, 2015; Strauss *et al*, 2014), it is tempting to predict that cortical GABA reduction in schizophrenia and depression produces common deficits in effort-based behavior through the decreased GABA levels in the ACC. In addition, in our mutant mice there were also changes in the circadian feeding pattern, which may be analogous to the night time eating syndrome sometimes observed in major depression (Gallant *et al*, 2012) and insomnia (Plante *et al*, 2012). Thus, these phenotypic domains elicited by lowered GABA levels are reminiscent of a subset of negative symptoms observed in human psychiatric disorders, which are not pathognomonic of schizophrenia but also appears frequently in depression.

## Funding and disclosure

This work was supported by the Intramural Research Program at the National Institute of Mental Health, National Institutes of Health (ZIA MH002895) and was funded in part by grants from the National Institute of Mental Health K22 MH099164 and R01 MH110681 (to K Nakazawa). The authors declare no conflict of interest.
