# Supplementary material for: Neuropsychiatric Phenotypes Produced by GABA Reduction in Mouse Cortex and Hippocampus
Source: Neuropsychopharmacology. 2018 Jan 24;43(6):1445–56. doi: 10.1038/npp.2017.296 (PMC5916365; doi:10.1038/npp.2017.296)
Supplement: Supplementary Information [file npp2017296x1.docx]

**SUPPLMENTARY INFORMATION**

**Neuropsychiatric phenotypes produced by GABA reduction in mouse cortex and hippocampus**

**Supplementary Methods**

All experimental procedures were approved by the Institutional Animal Care and Use Committee at National Institute of Mental Health and University of Alabama at Birmingham.

***Animals***

The generation and characterization of GABAergic interneuron-restricted Cre mice, *Ppp1r2*-Cre, was previously described (Belforte et al., 2010). Briefly, Cre expression began in the second postnatal week and was targeted strictly to ~50% of cortical and hippocampal interneurons (70% positive for parvalbumin (PV)). In the somatosensory cortex, about 70% of cre-targeted neurons are PV containing, and the rest includes Reelin-positive neurons, but negligible in the pyramidal neurons. Since Cre recombination occurs in over 80%, 75%, and 90% of PV neurons in the neocortex, mPFC and hippocampus, respectively, it is expected to confer efficient genetic manipulation in PV neurons. Cre expression is also detected in a majority of neurons of tenia tecta and lateral septum, and a subset of neurons in superior colliculus superficial layers. Cre recombination signals are also positive for 30-40% of PV neurons in dorsal striatum, while almost no PV neuron in ventral striatum are positive for Cre recombination. Negligible level of Cre recombination is detected in thalamic reticular nucleus (less than 5% of total) or ventral tegmental area (VTA)/ substantia nigra (SN) area (nearly 0%), or any other brain areas. These mice were crossed with *Gad1*-floxed mice (Chattopadhyaya et al., 2007) to generate conditional knockout of Gad67 mice targeted to this subset of interneurons (*Ppp1r2-Cre*^+/-^ : *Gad1*^loxP/loxP^), here after referred to as mutants, and their littermate *Gad1*-floxed control mice (*Ppp1r2-Cre*^-/-^ : *Gad1*^loxP/loxP^). All mice were maintained after backcrossing to C57BL/6 strain at least six times. For immunohistochemisty, the triple transgenic mice, *Ppp1r2-Cre^+/-^: Rosa26* ^loxP-Stop-loxP^*-EYFP^+/-^*: *Gad1*^loxP/loxP^ (referred to as Mutant-YFP) *and Ppp1r2-Cre^+/-^: Rosa26 ^loxP-Stop-loxP^-EYFP^+/-^: Gad1^+/+^* (referred to as Control-YFP) were used to visualize Cre recombination (Figure 1A).

***Immunohistochemistry***

Three slices containing mPFC, which includes anterior cingulate cortex (ACC) (three animals for each genotype, 10 weeks of age) were double-immunostained with chicken anti-GFP IgY, (1:500, GFP-1020, Aves Labs, OR, USA) and rabbit anti-GAD67 (1:500, ab97739, abcam, USA) and YFP/GAD67 co-localization on GAD67-positive interneurons in mPFC per genotype were quantified using confocal microscopy.

***Western blot***

Western blotting was performed as previously described (Rompala et al., 2013) using 10-12 week-old male mice, and quantified using ImageJ (NIH).

***Measurements of tissue amino acid contents***

Brain tissues of 11-13-week-old mice (6 male- and 8 female-floxed control mice vs 7 male- and 6 female-mutant mice), obtained following decapitation, were weighed and homogenized in 1.5 ml of methanol on ice. The homogenates were centrifuged at 3000×g for 6 min at 4°C, and 20 μl of supernatant was evaporated to dryness at 40°C. The residue was dissolved with a mixed solution of 70 μl H_2_O, 80 μl of 0.1 M borate buffer (pH 8.0), and 50 μl of 50 mM 4-fluoro-7-nitro-2,1,3-benzoxadiazole (NBD-F; Tokyo Kasei Kogyo Co., Ltd., Tokyo, Japan) in CH_3_CN. After 40 min at room temperature, 100 μl of 1 M tartrate buffer (pH 2.0) was added to stop the reaction. Quantification of amino acids was performed by a high-performance liquid chromatography (HPLC) system with fluorometric detection (Shimadzu Corporation, Kyoto, Japan) as previously described (Fujita et al., 2016). Since no sex difference was detected, male and female data were combined for analysis.

***Slice physiology***

To evaluate changes in inhibitory transmission in the anterior cingulate cortex (ACC), a part of medial prefrontal cortex (mPFC), whole cell patch clamp recording was performed (Gonzalez-Islas and Hablitz, 2001). Adult mice of either sex aged between 10 and 14 weeks were anesthetized using isofluorane and quickly decapitated. The brain was extracted and placed in ice-cold aCSF bubbled with carbogen and containing the following in mM: 110 choline, 2.5 KCl, 1.25 NaH_2_PO_4_.H_2_O, 0.5 CaCl_2_, 7 MgCl_2_, 10 dextrose, 25 NaHCO_3_, 1.3 ascorbate and 2.4 sodium pyruvate (Lobb et al., 2010). Coronal sections (350 µm thickness) containing the mPFC were cut using a Leica VT1000S vibratome and transferred to a holding chamber containing aCSF bubbled with carbogen containing in mM: 128 NaCl, 3 KCl, 10 dextrose, 0.5 ascorbate, 25 NaHCO_3_, 4 MgCl_2_ and 2 CaCl_2_. The slices were incubated at 36 °C for 45 min and then at room temperature for the remainder of the day.

The slice was placed in a perfusion chamber attached to the fixed-stage of an upright Olympus BX51WI microscope and submerged in continuously flowing oxygenated aCSF, which was similar in composition to the incubation aCSF except for MgCl_2_ at 1 mM. All recordings were performed at 32 °C. Pyramidal neurons in layer II/III of ACC were visualized with a 40× water-immersion lens and illuminated with near infrared (IR) light and the image was detected with an IR-sensitive CCD camera. The cells were identified based upon their large cone shaped soma and the presence of the apical dendrite towards layer I. Borosilicate patch pipettes (BF150-110-10, Sutter Instruments) of 3-5 MΩ resistance were pulled using a Narishige PC-100 puller and filled with the appropriate internal solution. For recording the evoked monosynaptic inhibitory postsynaptic currents (eIPSCs), the internal solution used contained in mM: 130 CsCl, 20 TEA, 10 HEPES, 1 EGTA, 2 Mg-ATP, 0.3 Na-GTP, 10 phosphocreatine and 5 QX-314, pH adjusted to 7.3 using CsOH and osmolarity adjusted to 285 mOsm. The cells were held at -70 mV during the recordings and glutamate transmission was blocked using the AMPA/KA receptor antagonist 6-cyano-7-nitroquinoxaline-2,3-dione (CNQX, 20 μM) and NMDA receptor antagonist d(−)-2-amino-5-phosphonopetanoic acid (D-APV, 50 μM). Stimulation (0.1 ms width, every 15 s) was delivered using a theta glass electrode (TG150-4, Warner instruments) filled with aCSF and an ISO-Flex stimulus isolator (A.M.P.I, Jerusalem, Israel). The stimulating electrode was placed approximately 100 µm from the soma of the recorded cell also in layer II/III, and increasing intensities of stimulation between 50 and 400 µA were applied. The IPSC amplitude was measured as the difference between the baseline prior to the stimulus artifact and the peak deflection occurring 2-4 ms after the stimulation.

Separate experiments to measure membrane properties of the neurons were done in current clamp mode using an internal solution containing in mM: 120 K-gluconate, 6 KCl, 4 Mg-ATP, 0.3 Na-GTP, 0.1 EGTA, 10 HEPES and 10 phosphocreatine, pH 7.3 using KOH, ~280 mOsm. Current was injected from -100 pA to 600 pA at 50 pA steps from resting membrane potential. Threshold, action potential (AP) width and amplitude, fast-after hyperpolarization (fAHP), input resistance and membrane time constant were measured offline using Clampex software. The data was collected with the help of a Multiclamp 700B amplifier (Axon Instruments, Union City, CA, USA) and 1322A A/D board, using the pClamp 10 program suite (Molecular Devices, Sunnyvale, CA), filtered at 3 kHz and digitized at 20 kHz. The access resistance was between 10 and 20 MΩ among different recorded neurons, and recordings were terminated if this value changed by over 20%.

To evaluate changes in inhibitory transmission and excitability in the hippocampus, whole cell patch clamp recordings were performed on CA1 pyramidal cells (Li et al., 2017). Adult mice (10-14 weeks; males and females) were anesthetized with isoflurane, decapitated, and brains quickly extracted. Four hundred-µm-thick coronal slices of hippocampus were cut on a vibrating microtome (VT1000S; Leica, Bannockburn, IL) and CA3 was removed. Slicing and dissection of the hippocampi were done in ice-cold (1–3°C) sucrose based dissection solution containing the following (in mM): 75 Sucrose, 87 NaCl, 3.0 KCl, 0.5 CaCl_2_, 7.0 MgCl_2_, 1.25 NaH_2_PO_4_, 26 NaHCO_3_, and 20 glucose, bubbled with 95% O_2_/5% CO_2_, pH 7.35–7.45. Slices recovered in a modified external recording solution (mERS). mERS contained the following compounds (in mM): 120 NaCl, 3.5 KCl, 0.75 CaCl_2_, 4.0 MgCl_2_, 1.25 NaH_2_PO_4_, 26 NaHCO_3_, and 10 glucose, bubbled with 95% O_2_/5% CO_2_, pH 7.35–7.45. During recovery, slices were incubated at 32 ⁰C for approximately 1 hr and then allowed to slowly cool to room temperature.

The hippocampal experiments were performed between 28°C to 30°C. During the experiments, slices were held in a submersion recording chamber perfused (2.5-3.5 mLs/min) with external recording solution (ERS). ERS contained the following compounds (in mM): 120 NaCl, 3.5 KCl, 2.5 CaCl2 and 1.3 MgCl2, 1.25 NaH2PO4, 26 NaHCO3, and 10 glucose, bubbled with 95% O2/5% CO2, pH 7.35–7.45. CA1 pyramidal cells were blindly patched on a Zeiss Examiner A1. Patch electrodes (4– 6MΩ) were filled with internal solution composed of the following (in mM): 120 K-gluconate, 2.6 KCl, 1.3 NaCl, 10 HEPES, 0.1 EGTA, 10 ATP-Na, 0.3 GTP, and 5 phosphocreatine-Tris, pH was adjusted to 7.2.

To isolate monosynaptic inhibitory postsynaptic currents (IPSCs) 10 μM NBQX (2,3-Dioxo-6-nitro-1,2,3,4-tetrahydr­obenzo[f]quinoxaline-7-sulfonamide) and 50 µM D-APV (D-2-amino-5-phosphonopentanoic acid) were added to the ERS. CA1 pyramidal cells were patched in the voltage-clamp configuration and held at 0 mV using a Multiclamp 700A amplifier (Molecular Devices, Sunnyvale, CA). The access resistance and holding current (<200 pA) were monitored continuously. Recordings were rejected if either access resistance or holding current increased >25% during the experiment. eIPSCs were recorded in response to extracellular stimulation elicited using a bipolar tungsten microelectrode (FHC, Bowdoinham, ME). Stimulation was generated from a Master-9 digital stimulator (A.M.P.I.) and applied with a BSI-2 biphasic stimulus isolator (BAK Electronics, Mount Airy, MD). The maximal eIPSC was determined and stimulation was reduced to generate a response whose amplitude was 40 to 60% of the maximum eIPSC to obtain a stable baseline. The stimulation intensity ranged from 10 µA to 250 µA with a duration of 100 µs. The stimulating electrode was placed in *s. pyramidale* to stimulate axons from somatic targeting GABAergic neurons. The stimulus intensity of the threshold evoked response, referred to as 1x, was determined as the intensity which gave a response between 10 to 15 pA. Stimulation was increased in a multiplicative manner to develop the input-output curve. We observed reduced IPSCs when the internal recording solution contained both high chloride (Figure 1E) and lower chloride (Figure S2A), indicating that the effect was not dependent on the intracellular chloride level.

In separate experiments, intrinsic excitability of CA1 pyramidal cells was measured at holding potential of -60 mV in current clamp mode. 600 ms current steps (0–400 pA, in 50 pA intervals; 400-800 pA in 100 pA intervals) were applied every8 s, and the initial firing frequency (based on interval between the first three spikes) and total number of spikes were measured. These recordings were done in ERS only. In a separate cohort of animals, the intrinsic excitability of CA1 pyramidal cells was measured in the presence of 100 µM picrotoxin and 10 µM CGP55845 to block GABAA and GABAB receptors, respectively.

***In vivo microdialysis*** ***under tail suspension test or amphetamine treatment***

The mice (12-20 weeks old, mixed gender) were anesthetized 2% isoflurane, mounted in a stereotaxic instrument (model 963, David Kopf Instruments, Tujunga, CA) and implanted with a unilateral microdialysis guide cannula (CMA7 Guide Cannula, CMA Microdialysis AB, Sweden) aimed at the anterior cingulate cortex (ACC) (+1.8 mm AP, -0.3 mm ML from bregma and -1.5 mm from brain surface (DV)) or the lateral shell of nucleus accumbens (NAC) (+1.0 mm AP, -2.0 mm ML from bregma and -2.8 mm DV from brain surface). Twentyfour to 48 hr after the surgery, a microdialysis probe (CMA7) was inserted through the guide cannula and connected to a dual-channel stainless steel swivel (375/D/22, Instech Laboratories, Inc., PA, USA) and then a mouse was placed into a microdialysis test chamber (30-cm diameter and 38-cm height). Probes were perfused at 1 µl/min with aCSF (0.13 M NaCl, 4 mM KCl, 0.75 mM NaH_2_PO_4_, 2mM Na_2_HPO_4_, 2 mM MgCl_2_, 1.7 mM CaCl_2_) for 2 hr using a 2.5 ml gas-tight syringe (Microsyringe 2.5 mL glass, CMA Microdialysis AB, Sweden) and syringe pump (CMA 402 Syringe Pump, CMA Microdialysis AB, Sweden) (Tejeda et al., 2013). Following a 2-hr equilibration period, samples were collected every 20 min at 1 µl/min perfusion rate into a microcentrifuge tube added 1.5 µl of 0.1M CH_3_COOH including 2.0 mM (COOH)_2_ and 6.0 mM L-Cysteine. Samples were collected 3 times (each 20-min long) prior to injecting the mice with d-amphetamine (2.5 mg/kg, *i.p.*) or tail-suspension test (TST) for 6 min and samples were collected 4 more times after the treatment. For TST, mice were suspended 30 cm above the floor by adhesive tape placed approximately 2 cm from the tip of the tail while sample was collected. To prevent the tendency of C57BL/6 mice to climb their tails, a clear plastic cylinder (4-cm long, 1.5-cm in diameter) was placed around their tails, as described (Can et al., 2012). Mice behavior was monitored using a camera with keeping the dialysate sampling during TST paradigm. After TST, the mouse was put back to the chamber, and samples were continually collected every 20 min. Following the experiment, all samples were frozen at -80°C and mouse brains were fixed in 4% paraformaldehyde and sectioned (50-µm thickness), stained with cresyl violet, and examined for verification of probe placement. Dopamine and Serotine dialysate concentrations were analyzed using high performance liquid chromatography (HPLC) with electrochemical detection (Eicom, HTEC-500 HPLC/EC system, Kyoto, Japan). Dialysates (20 µl each from ACC and 10 µl each from NAC) were injected into a HPLC system with an integrated amperometric detector, consisting an ODS HPLC column (Eicom, PP-ODS2, Kyoto, Japan), a HTEC-500 pump and working electrode (Graphite, WE-3G, Eicom, Kyoto, Japan) with a Ag/AgCl reference electrode (Vapplied = +400 mV). The mobile phase contained 1.5% MeOH, 100 mM phosphate buffer, pH 5.4, 500 mg/L of sodium 1-Decanesulfonate (DSS), and 50 mg/L of EDTA-Na2. Envision Chromatography Suite (Eicom, Kyoto, Japan) was used to acquire and analyze chromatographic data.

***In vivo microdialysis under progressive ratio responding (PR) test and subsequent tail pinch***

A hand-made pellet receptacle with 3-cm tall opening was attached to the murine operant conditioning chamber (Med Associates Inc., Fairfax, VT) to make the probe-implanted mice accessible to the pellets. After the animal was trained in the fixed-ratio (FR) schedules of reinforcement (see behavioral method section), the mice were implanted with a unilateral microdialysis guide cannula aimed at the anterior cingulate cortex (ACC) (+1.8 mm AP, -0.3 mm ML from bregma and -1.5 mm DV). Three days after the surgery, the animal was retrained with FR-5 schedule of reinforcement. On the following day, a microdialysis probe was inserted to ACC through the guide cannula and the animal was placed into a microdialysis test chamber (30-cm diameter and 38-cm height). Following a 2-hr equilibration period, dialysate samples were collected 3 times (each 20-min bin) as a baseline data prior to PR responding test at 1 µl/min perfusion rate. Then, the animal was placed into the operant chamber and samples were collected furthermore during PR responding test (80-min total). When the microdialysis tube was twisted, the door of operant chamber was opened and the tube was untwisted to allow the animal to move freely. After PR responding test, the animal was put the microdialysis test chamber again and pinched the tail for 1 min using halstead mosquito forceps (RS-7112, Roboz, USA), and dialysate samples were collected 2 more times (20-min bin each) after the tail pinch.

***In vivo electrophysiology***

To assess the epileptiform activity in the cortex, local field potential (LFP) recordings from the primary sensory cortex were conducted during a 10-min immobility session in a high-walled small circular box (10-cm in diameter)(Jinde et al., 2009). Subsequently, they were also subjected to a simple alternation task for 10 min in a low-walled linear track (length, 72 cm; width, 8 cm) for LFP recording during exploratory behavior. When the mice stop exploration during 10 min, they were touched by a thin stick and forced to explore. Recordings were repeatedly conducted at 10, 12, and 15 weeks of age from the same animal.

***Behavioral testing***

Mice were single-housed for at least one-week prior to behavioral testing and used at 10-12 weeks of age, unless otherwise noted. Housing was maintained on a 12-hr light/dark cycle (light 6 am to 6 pm) with food and water ad libitum. All tasks, except for the spatial Y-maze tasks, were performed during the light phase by an investigator blind to genotype and treatment. Independent cohort of animals was used for most of tasks, except open field test and light/dark test. All apparatuses were cleaned with 70% ethanol between animals during testing.

*Light/Dark Box*

The light/dark apparatus consists of a 2-chamber box (75 x 30 cm, l x w; 7-cm high passage between chambers) – one brightly lit (>300 lux) and the other dark (<5 lux). During testing, the mice were placed in the dark chamber and their behavior was recorded for 10 min, as described (Bourin and Hascoët, 2003). The total time in the light side was considered an index of anxiety or risk-avoidance.

*Sociability test*

Sociability was measured in a three-chamber box in which one side chamber contained a wire mesh cage with a novel conspecific mouse and the other side chamber contained an empty wire-mesh cage. Habituation and testing took place as previously described (Silverman et al., 2010; Yang et al., 2001). Time the mice spent with their noses within 3 cm of the wire cages were recorded as investigation time by Any-Maze (Stoelting Co., Wood Dale, IL) software.

*Female Interaction (chasing)*

Super-ovulating female mouse (6-8 weeks old) was placed in the home cage of male mouse. Their interactions (chasing) were video-recorded for 8 min and analyzed as described (Silverman et al., 2010). To induce super-ovulation in wild-type C57BL/6 female mice, three days before testing they were injected with pregnant mares' serum and then 24 hr prior to testing they received human chorionic gonadotropin injections.

*Home-cage Wheel Running*

Running-wheel activity was recorded and analyzed with ClockLab (Actimetrics, Wilmetter, IL) (Goh and Ladiges, 2011). Mice were placed at 6:00pm (start of dark cycle) in individual cages equipped with running-wheels and housed in ventilated boxes with access to food and water. During light-on, the room was well illuminated (100 lux). The 12-hr light/dark cycle was maintained with the same schedule as in the home-cage. Mice were maintained in the running wheel cages and recording was continuous for 96 -120 hr. For purpose of analysis, the first 24 hr were not included.

*Tail-suspension test (TST)*

Mice were suspended by a 17-cm length of tape (TimeMed Labeling Systems, Inc., Burr Ridge, IL). Mobility was defined as movement of the hind legs and an observer blind to genotype scored the recorded video for six min. To prevent the tendency of C57BL/6 mice to climb their tails, a clear plastic cylinder (4 cm length, 1.5 cm diameter) was placed around their tails, as described (Can et al., 2012).

*Learned Helplessness*

Training and testing took place in the Gemini avoidance system (San Diego Instrument, San Diego, CA) using methods similar to those previously reported with some modifications (Shirayama et al., 2002). During training 120 inescapable shocks were delivered [15-sec duration, randomized 45-sec inter-stimulus interval (ISI)]. During testing, the shock was terminated when the animal crossed between the two chambers or after 24 sec. One presentation of cue light/tone and shock was considered a single trial. Testing consisted of 30 trials (Inter-trial interval, 22-38 sec). The number of escapes during testing was considered an index of learned helplessness.

*Operant Conditioning under progressive ratio schedule of reinforcement*

Experimenters used standard operant chambers for mice (16 × 14 × 12.5 cm) with grid floors controlled by Med-PC IV software (Med Associates Inc., Fairfax, VT). Each chamber was housed in a sound-attenuating outer chamber equipped with a white noise generator and fan. A house light illuminated the chamber, and a dispenser delivered grain-based food pellets (20 mg; Bio-Serv, Frenchtown, NJ) into the magazine. Head entries into the stimuli and magazine were detected by photocell.

Mice were initially allowed to consume reinforcer pellets in their home cages for 1 day. Mice were then habituated to the testing apparatus with 15 min unlimited food pellet access. Next, subjects were trained to nose-poke on a fixed ratio (FR)-1 reinforcement schedule whereby a single nose-poke elicits the delivery of a food pellet to the receptacle, with a 5-sec time out (TO). Each FR training session lasted 12 hr or when 120 pellets have been delivered. Mice exhibiting discrimination of ≥3:1 for the active versus inactive holes and obtaining ≥ 80% of all available pellets (out of 120 pellets) per session over three consecutive sessions were considered to achieve acquisition criteria. Then, the schedule was increased to FR5/TO-5 seconds in which 5 active nose-poking trigger the delivery of the food pellet. Training on the FR5 schedule lasted three days. The position of the active nose poke (left, right, or center) was counterbalanced between and within groups. Mice were required to display stable responding for 3 consecutive days to complete the acquisition phase. All mice regardless of genotypes acquired the task within 7 days (data not shown).

The mice are then subjected to the progressive ratio schedule of reinforcement. The response ratio schedule during progressive ratio testing can be calculated as per Richardson and Roberts (Richardson and Roberts, 1996) using the following formula (rounded to the nearest integer): = [5e (R*0.2)] – 5, where R is equal to the number of food rewards already earned plus 1 (i.e., next reinforcer). Thus, the number of responses required to earn a food reward follow the order: 1, 2, 4, 6, 9, 12, 15, 20, 25, 32, 40, 50, 62, 77, 95, 118, 145, 178, 219, 268, 328, 402, 492, 602 and so on. The final ratio (max. required poking response number) completed is the breakpoint. A progressive ratio responding session lasts a maximum of 1 hr. Failure to nose-poke in any 10 min period results in termination of the session. Performance on the progressive ratio schedule of reinforcement is considered stable when the number of rewards earned in a 1 hr session deviates by ≤10% for at least 3 consecutive days.

*Automated Homecage Scan*

Using automated video analysis software (Homecage Scan, CleverSys, Inc., Reston, VA) we recorded a number of baseline behaviors over a continuous and undisrupted 48-hr period, as previously described (Adamah-Biassi et al., 2013). By utilizing animal body part classifiers, sequence data was able to automatically recognize behaviors including total activity, eating, drinking, grooming, walking, rearing, and twitching. The mice were maintained in their normal homecages within a sound attenuating chamber that allowed for controlled temperature, ventilation, lighting (150-lux during light cycle and infrared illumination during dark cycle) and a white noise background.

*Other Behavioral Tasks*

All other behavioral tasks, including the open field test, rotarod test, mating test, forced swim test, saccharine preference, prepulse inhibition (PPI) of acoustic startle reflex, Y-maze spontaneous alternation test, MK-801 and amphetamine locomotor responses, and nest-building test were performed as previously described (Belforte et al., 2010).

***Statistical analysis***

Data were analyzed using *Student*’s t-test, paired t-test, or a factorial model analysis of variance (ANOVA) using genotype and cohort as “between subjects,” and times of testing as “within subject” (repeated measure). *Post-hoc* analyses were performed using *Bonferroni* corrections. Differences were considered to be significant when p< 0.05 (two tailed).

**Supplementary Discussion**

The deletion of *Gad1* in approximately 50% of cortical and hippocampal interneurons in the mutant mice resulted in a 50% decrease in GAD67 protein. This was accompanied by an increase in GAD65, as has previously been observed in some mice with *Gad1* deletion (Fujihara et al., 2015; Georgiev et al., 2016). Measurement of tissue GABA levels showed significant decreases in cortical areas in the mutants, indicating that the enhancement of GAD65 cannot fully compensate for the loss of GAD67. Importantly, this resulted in significant decrease in the strength of GABAergic transmission onto pyramidal cells in both mPFC and hippocampus. This is consistent with the reduction in GABA IPSC amplitude previously observed with heterozygous deletion of *Gad1* specifically from parvalbumin cells in hippocampus (Fujihara et al., 2015) and mPFC (Brown et al., 2015; Lazarus et al., 2015). The impairment of GABAergic transmission in the mutants is likely to be caused by reduced GABA per vesicle (Lau and Murthy, 2012), along with possible reduction in GABAergic synapse number (Chattopadhyaya et al., 2007).

Mutant mice also showed a moderate enhancement in intrinsic excitability of pyramidal cells, as seen as an increase in action potential number in response to depolarizing current steps, both in mPFC and in hippocampus. This was no longer observed when GABAergic synaptic transmission was blocked, indicating that it is secondary to the GABAergic deficit. However, this unmasked a small decrease in the initial firing frequency, which was not observed in the absence of GABA receptor antagonists, suggesting that there is postsynaptic compensation in mutants that reduces the initial firing frequency. Interestingly, this means that the postsynaptic adaptations are able to partially compensate for the GABAergic deficit, by preventing the change in initial firing frequency, while causing a change in sustained firing during the current steps. Changes in intrinsic excitability have previously been observed in response to GAD67 deficiency and a dependence on GABAergic transmission was verified (Lazarus et al., 2015). In contrast, we saw no difference in action potential threshold between mutants and controls, with or without GABA receptors blocked. This indicates that alterations in GABAergic transmission do not change the action potential threshold, consistent with a previous study that showed no effect on action potential threshold of exogenously applied GABA or gabazine (Torres-Torrelo et al., 2014).


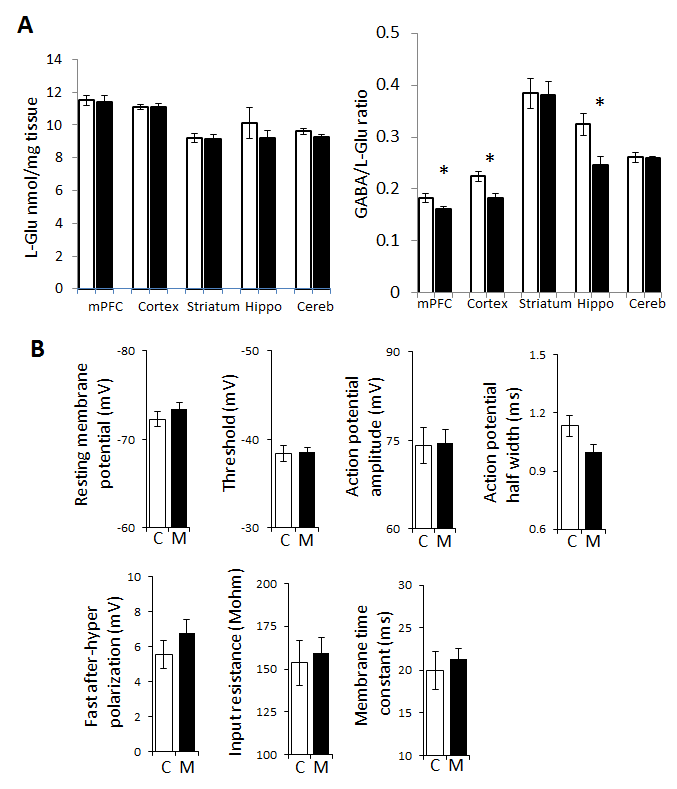


**Figure S1**

**A**, Tissue L-glutamate levels are unaltered by *Gad1* deletion in any brain regions, Accordingly, GABA/L-glutamate ratio decreases in mPFC, frontal cortex, and hippocampus. t(25)= -2.12, **p*=0.04 for mPFC; t(25)= -3.15, **p*=0.004 for frontal cortex(Cortex); t(25)= -2.87, **p*=0.008 for hippocampus (Hippo). n=14 for floxed-control (white), n=13 for mutant mice (black).

**B**. We investigated whether the *Gad1* deletion causes changes in the excitability of pyramidal neurons in ACC. By injecting a series of steps hyper- and depolarizing current steps into the soma of the recorded neuron from resting membrane potential, action potentials were evoked and the membrane properties were measured. All cells recorded showed regular spiking and adapting action potentials. Threshold, action potential half-width and amplitude, fAHP(fast-after hyperpolarization), input resistance and membrane time constant did not vary significantly between the two groups. However, the neurons in the mutant group were more excitable as indicated by the higher number of action potentials fired (**Figure 1E**) indicating that the pyramidal neurons in the mutant mice may be disinhibited due to impairment of GABAergic transmission.


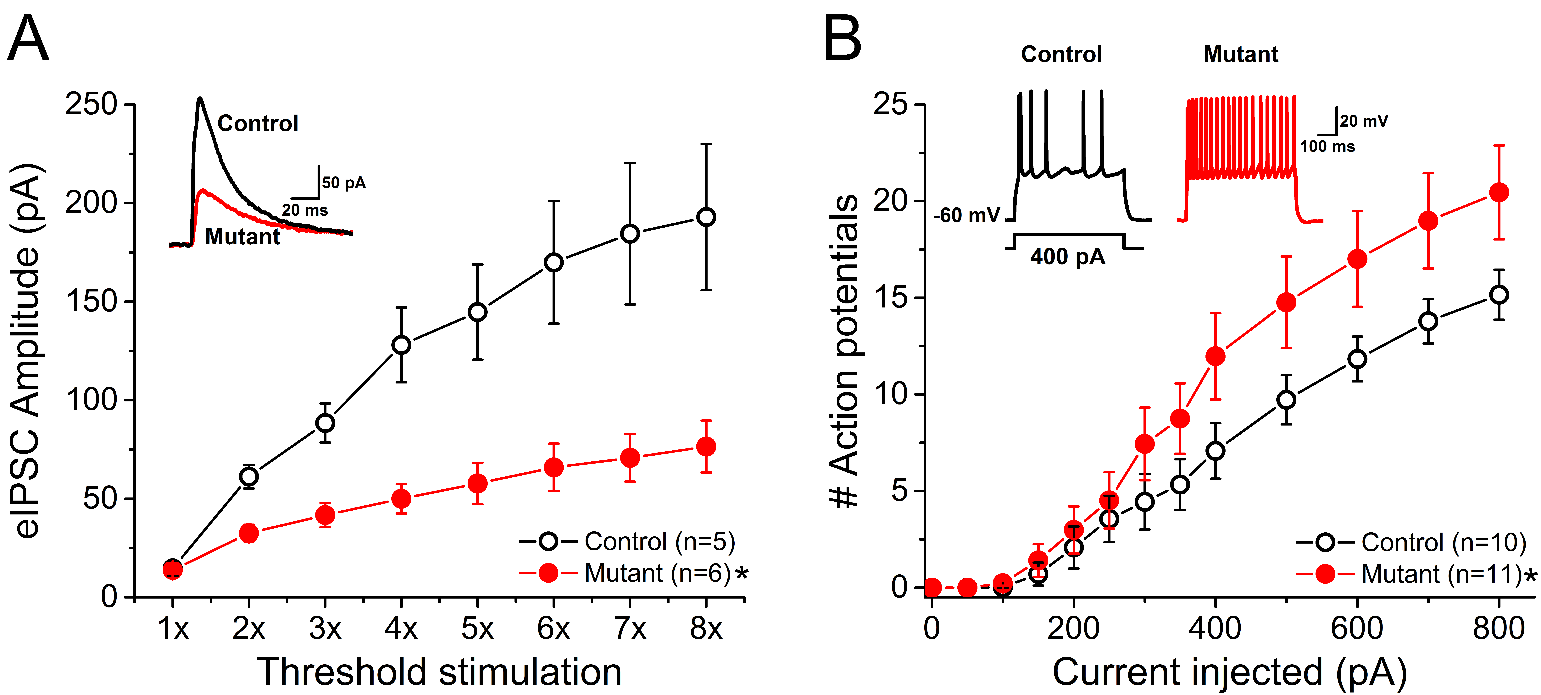


**Figure S2,** **A** Evoked monosynaptic IPSC amplitudes measured in CA1 pyramidal cells in response to stimulation of s. pyramidale are dramatically reduced in hippocampal slices from mutant mice (Two-Way ANOVA, main effect of genotype, F_(1,72)_=70.63, **p*<0.0001 N=5 for floxed-controls, N=6 for mutants). eIPSCs were measured in the presence of 10 µM NBQX and 50 µM D-APV. Inset: example trace of monosynaptic eIPSCs from control (black) and mutant (red) mice. **B** Action potential numbers are higher in the mutant CA1 pyramidal neurons (Two-Way ANOVA, main effect of genotype, F_(1,243)_=21.01, **p*<0.0001) indicating increased excitability during sustained firing. There was no change in the initial firing frequency (data not shown). N=10 for floxed controls, N=11 for mutants. Inset: Example recordings showing the firing patterns of CA1 pyramidal cells in response to 400 pA current step from control (black) and mutant (red) mice. All firing data were measured without synaptic blockers in the recording solution.


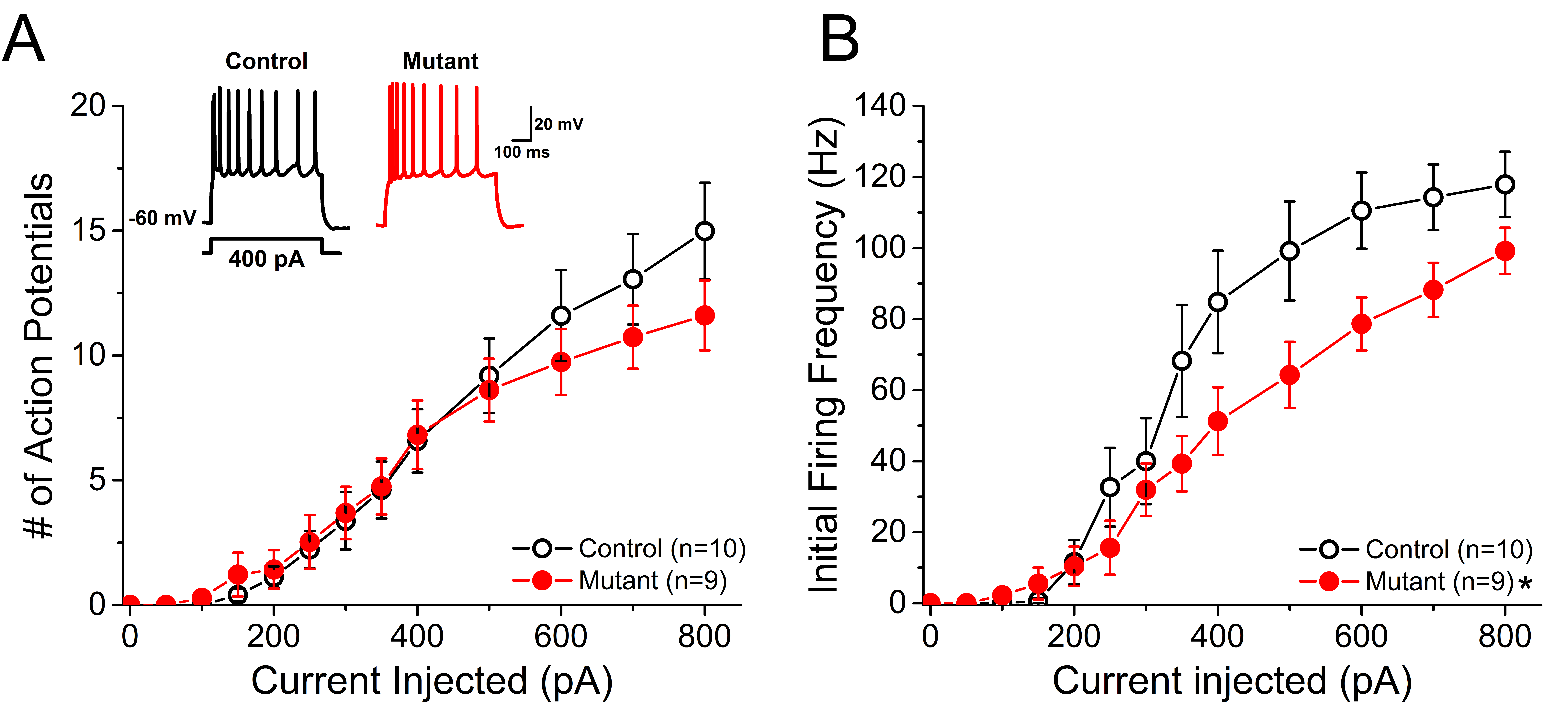


**Figure S3,** **A** In the presence of GABA_A_ and GABA_B_ receptor antagonists (100 µM picrotoxin and 10 µM CGP55845), the action potential numbers are not different between mutant and control CA1 pyramidal neurons (Two-Way ANOVA, main effect of genotype, F_(1,221)_=1.04, p=0.31, N=10 for floxed controls, N=9 for mutants). Inset: Example recordings showing the firing patterns of CA1 pyramidal cells in response to 400 pA current step from control (black) and mutant (red) mice. **B**, Blocking GABA_A_ and GABA_B_ receptors reveals a change in the initial firing frequency between the genotypes. The initial firing frequency was decreased in the mutant CA1 pyramidal neuron, indicating decreased intrinsic excitability (Two-Way ANOVA, main effect of genotype, F_(1,221)_=19.72, **p*<0.0001, N=10 for floxed controls, N=9 for mutants).

**Figure S4**, **A & B**. *Gad1* ablation did not impact any measures of progressive ratio responding, including the total number of nose poking during 12-h session (**A,** t(16)=0.14, p= -0.89), or average rate of nose poking during the course of the session (**B,** Repeated measures ANOVA, F(1,258)=1.19, p=0.27). **C.** The overall pattern of the male mutant behaviors in their homecage did not differ significantly from that of the controls over 48 h. There was a trend towards the mutants being more stationary (p=0.08).

**Figure S5**, Coronal sections showing microdialysis probe locations measured from bregma, using the Paxinos and Franklin’s Mouse Brain in Stereotaxic Coordinates, 4^th^ Edition (2013). (**A**) Microdialysis probes were inserted in the anterior cingulate cortex (ACC) for tail suspension (black) or for progressive ratio (PR) responding test (blue). Pictures indicate that the dialysates are collected from the behaving animal during the tail suspension (upper) or PR responding tests (lower). (**B**) Microdialysis probes were inserted into nucleus accumbens (NAC) lateral shell region for tail suspension (black) or for amphetamine challenge (red).

**Figure S6,** Representative examples of LFPs from somatosensory cortex and hippocampus in control mouse while mouse was held immobile in a small chamber (10-cm diameter). No interictal discharges, which were synchronized across cortical and hippocampal electrodes, were detected at any recording periods from all the mice tested (n=5).

**Figure S7.** (**A**) Tail suspension did not increase NAC dopamine level in either controls (paired t-test between -20-0 min and 0-20 min, t(4)= -1.75, p=0.15) or mutants (paired t-test between -20-0 min and 0-20 min, t(5)= -2.03, p=0.097). Accordingly, no genotypic difference in NAC dopamine levels after tail suspension (repeated measures ANOVA, F(1,27)=1.78, p=0.17, post-hoc planned comparisons, at 20-40 min; F(1,9)=2.99, p=0.11). (**B**) Controls (paired t-test between -20-0 min and 0-20 min, t(9)= -3.16, p=0.011) and mutants (paired t-test between -20-0 min and 0-20 min, t(3)= -4.65, p=0.018) both slightly increased 5-HT levels in NAC by tail suspension. However, there was no genotypic difference in the degree of NAC 5-HT increase after tail suspension (repeated measures ANOVA, F(1,24)=0.08, p=0.97). (**C**) Individual animal data of Figure 5E also showed an increase in ACC dopamine during progressive ratio responding regardless of genotypes. (**D**) No difference in the progressive ratio breakpoints between genotypes of the animals assessed in Figures 5E and S7C, t(14)=0.45, p=0.66. Data are mean ± s.e.m.; n is indicated in parentheses or plot bars.

**REFERENCES**

Adamah-Biassi, E. B., Stepien, I., Hudson, R. L., and Dubocovich, M. L. (2013). Automated video analysis system reveals distinct diurnal behaviors in C57BL/6 and C3H/HeN mice. *Behavioural Brain Research* **243**, 306-312.

Belforte, J. E., Zsiros, V., Sklar, E. R., Jiang, Z., Yu, G., Li, Y., Quinlan, E. M., and Nakazawa, K. (2010). Postnatal NMDA receptor ablation in corticolimbic interneurons confers schizophrenia-like phenotypes. *Nat Neurosci* **13**, 76-83.

Bourin, M., and Hascoët, M. (2003). The mouse light/dark box test. *European Journal of Pharmacology* **463**, 55-65.

Brown, J. A., Ramikie, T. S., Schmidt, M. J., Baldi, R., Garbett, K., Everheart, M. G., Warren, L. E., Gellert, L., Horvath, S., Patel, S., and Mirnics, K. (2015). Inhibition of parvalbumin-expressing interneurons results in complex behavioral changes. *Mol Psychiatry* **20**, 1499-507.

Can, A., Dao, D. T., Terrillion, C. E., Piantadosi, S. C., Bhat, S., and Gould, T. D. (2012). The tail suspension test. *J Vis Exp*, e3769.

Chattopadhyaya, B., Di Cristo, G., Wu, C. Z., Knott, G., Kuhlman, S., Fu, Y., Palmiter, R. D., and Huang, Z. J. (2007). GAD67-mediated GABA synthesis and signaling regulate inhibitory synaptic innervation in the visual cortex. *Neuron* **54**, 889-903.

Fujihara, K., Miwa, H., Kakizaki, T., Kaneko, R., Mikuni, M., Tanahira, C., Tamamaki, N., and Yanagawa, Y. (2015). Glutamate Decarboxylase 67 Deficiency in a Subset of GABAergic Neurons Induces Schizophrenia-Related Phenotypes. *Neuropsychopharmacology* **40**, 2475-86.

Fujita, Y., Ishima, T., and Hashimoto, K. (2016). Supplementation with D-serine prevents the onset of cognitive deficits in adult offspring after maternal immune activation. *Sci Rep* **6**, 37261.

Georgiev, D., Yoshihara, T., Kawabata, R., Matsubara, T., Tsubomoto, M., Minabe, Y., Lewis, D. A., and Hashimoto, T. (2016). Cortical Gene Expression After a Conditional Knockout of 67 kDa Glutamic Acid Decarboxylase in Parvalbumin Neurons. *Schizophr Bull* **42**, 992-1002.

Goh, J., and Ladiges, W. (2011). Voluntary Wheel Running in Mice. *In* "Current Protocols in Mouse Biology". John Wiley & Sons, Inc.

Gonzalez-Islas, C., and Hablitz, J. J. (2001). Dopamine inhibition of evoked IPSCs in rat prefrontal cortex. *J Neurophysiol* **86**, 2911-8.

Jinde, S., Belforte, J. E., Yamamoto, J., Wilson, M. A., Tonegawa, S., and Nakazawa, K. (2009). Lack of kainic acid-induced gamma oscillations predicts subsequent CA1 excitotoxic cell death. *Eur J Neurosci* **30**, 1036-55.

Lau, C. G., and Murthy, V. N. (2012). Activity-dependent regulation of inhibition via GAD67. *J Neurosci* **32**, 8521-31.

Lazarus, M. S., Krishnan, K., and Huang, Z. J. (2015). GAD67 deficiency in parvalbumin interneurons produces deficits in inhibitory transmission and network disinhibition in mouse prefrontal cortex. *Cereb Cortex* **25**, 1290-6.

Li, Q., Bartley, A. F., and Dobrunz, L. E. (2017). Endogenously Released Neuropeptide Y Suppresses Hippocampal Short-Term Facilitation and Is Impaired by Stress-Induced Anxiety. *J Neurosci* **37**, 23-37.

Lobb, C. J., Wilson, C. J., and Paladini, C. A. (2010). A dynamic role for GABA receptors on the firing pattern of midbrain dopaminergic neurons. *J Neurophysiol* **104**, 403-13.

Richardson, N. R., and Roberts, D. C. (1996). Progressive ratio schedules in drug self-administration studies in rats: a method to evaluate reinforcing efficacy. *J Neurosci Methods* **66**, 1-11.

Rompala, G. R., Zsiros, V., Zhang, S., Kolata, S. M., and Nakazawa, K. (2013). Contribution of NMDA receptor hypofunction in prefrontal and cortical excitatory neurons to schizophrenia-like phenotypes. *PLoS One* **8**, e61278.

Shirayama, Y., Chen, A. C., Nakagawa, S., Russell, D. S., and Duman, R. S. (2002). Brain-derived neurotrophic factor produces antidepressant effects in behavioral models of depression. *Journal of Neuroscience* **22**, 3251-61.

Silverman, J. L., Yang, M., Lord, C., and Crawley, J. N. (2010). Behavioural phenotyping assays for mouse models of autism. *Nat Rev Neurosci* **11**, 490-502.

Tejeda, H. A., Counotte, D. S., Oh, E., Ramamoorthy, S., Schultz-Kuszak, K. N., Backman, C. M., Chefer, V., O'Donnell, P., and Shippenberg, T. S. (2013). Prefrontal cortical kappa-opioid receptor modulation of local neurotransmission and conditioned place aversion. *Neuropsychopharmacology* **38**, 1770-9.

Torres-Torrelo, J., Torres, B., and Carrascal, L. (2014). Modulation of the input-output function by GABAA receptor-mediated currents in rat oculomotor nucleus motoneurons. *J Physiol* **592**, 5047-64.

Yang, M., Silverman, J. L., and Crawley, J. N. (2001). Automated Three-Chambered Social Approach Task for Mice. *In* "Current Protocols in Neuroscience". John Wiley & Sons, Inc.
